# One-year continuation of postpartum intrauterine contraceptive device: findings from a retrospective cohort study in India^☆^^☆☆^

**DOI:** 10.1016/j.contraception.2018.12.003

**Published:** 2019-04

**Authors:** Somesh Kumar, Ashish Srivastava, Surendra Sharma, Vivek Yadav, Atul Mittal, Young–Mi Kim, Angela Nash-Mercado, Sijmen A. Reijneveld, Bulbul Sood

**Affiliations:** a Jhpiego, an affiliate of Johns Hopkins University; b University Medical Center Groningen, University of Groningen, Department of Health Sciences

**Keywords:** Postpartum, Family planning, Intrauterine contraceptive device, Long acting reversible contraceptives

## Abstract

**Objective:**

To evaluate outcomes of a national postpartum (within 48 h of delivery) copper intrauterine device placement (PPCuIUD) program in six “high-focus states” with high unmet family planning need in India.

**Study design:**

We identified high-volume district hospitals that provided PPCuIUD in six (Bihar, Jharkhand, Uttar Pradesh, Uttarakhand, Madhya Pradesh and Chhattisgarh) Indian states (two per state). Each selected hospital maintained a list of PPCuIUD acceptors with contact phone numbers. We randomly selected 100 women at each site for inclusion in a telephone survey of IUD outcomes at 1 year. Questions regarded IUD expulsion, discontinuation because of symptoms (e.g., pain, bleeding, discharge), discontinuation for other reasons and use of alternative contraception if discontinuation reported.

**Results:**

We could contact 844 of the 1200 randomly selected women, of whom 673 (79.7%) had postplacental insertion (within 10 min of delivery), while 171 (20.3%) had an early postpartum insertion (between 10 min to 48 h after delivery). Of those contacted, 530 women (62.8%) reported continuing with the method beyond 1 year, 63 (7.5%) reported having an expulsion, 163 (19.3%) reported having removals for associated side effects (bleeding, pain and discharge), and 88 (10.4%) reported having removals for other reasons. After removal or expulsion, almost half of the women (46.5%) did not switch to any other modern contraceptive method.

**Conclusion:**

PPCuIUD continuation rate at 1 year was 62.8%. Most removals within 1 year were due to associated side effects. Almost half of the women discontinuing PPCuIUD did not switch to an alternative modern contraceptive method.

**Implications:**

The 1-year continuation rate of PPCuIUD achieved through a large-scale national program in India is satisfactory. The program though needs to address the low uptake of other modern contraceptive methods after discontinuation.

## 1 Introduction

India's longstanding Family Planning (FP) program, which started in 1952 [1], has traditionally focused on limiting family size by female sterilization, with more than 50% of FP users relying on them [2]. The program has had limited focus on further aspects of family planning. This highlights the need to increase awareness of and access to spacing methods. Recognizing these needs, the Government of India has undertaken multiple strategies for increasing access to effective methods for spacing births [1].

A central component of these strategies is to strengthen access to postpartum FP services for women of reproductive age. As a part of these efforts, the Indian Government, with technical assistance from Jhpiego, introduced postpartum intrauterine contraceptive device (PPCuIUD) services in 2010–2011 [3]. This was based on global evidence that PPCuIUD is a safe and effective contraceptive method [4], [5] and contact with the health system during childbirth provides an opportunity to make FP services accessible to women in resource constrained settings [6].

After the introduction, India rapidly scaled up its PPCuIUD program, which resulted in more than 2.8 million women choosing PPCuIUD over the last 6 years [7]. Other countries have recently reported similar expansions of PPCuIUD services [6]. The rapid expansion of PPCuIUD — in India as well as globally — calls for more evidence on the outcomes of these programs in terms of continuation rates at 1 year and beyond. While global evidence on continuation rates is available for interval intrauterine contraceptive device (IUCD) insertions [8], [9], [10], [11], [12], [13], [14], [15], [16], [17], it is limited for PPCuIUDs. The few studies on PPCuIUD continuation rates are restricted to hospital settings [18], [19], [20], but there is little evidence from any program implemented at scale [21]. Considering the complexities in scale-up of any new intervention in resource-constrained health systems, this lack of program level evidence is a barrier to informed decision making for scale-up of these services globally.

To address this need, we designed a retrospective single-arm cohort study — within a large scale PPCuIUD program — to determine the 1-year continuation rate and reported complication rates, including rates of expulsion of PPCuIUDs. We also assessed the reported reasons for discontinuation — through removal and expulsion — of PPCuIUDs within 1 year as well as the contraceptive switching choices among women who discontinued PPCuIUDs.

## 2 Methods

### 2.1 Sample

We conducted this retrospective single-arm cohort study in November–December 2015 in six (Bihar, Jharkhand, Uttar Pradesh, Uttarakhand, Madhya Pradesh and Chhattisgarh) of India's 36 states which are considered “high-focus states” due to poor health indicators including high unmet need for FP.

For sample size estimation, we hypothesized the 1-year continuation rate of PPCuIUD to be 60%. This is a conservative estimate based on the findings (76%) of a previous study [22]. We took a conservative estimate considering our study was being done within a large-scale national program. Aiming at determining this estimate with a precision of ±6%, i.e., a confidence interval (CI) of 12%, multilevel design effect of 2 (to account for clustering of study participants by district hospitals) and a possible nonresponse/refusal rate of 50%, we calculated a sample size of 1016. We rounded it to 1200 to equally distribute the sample across 12 district hospitals.

**Fig. 1:**
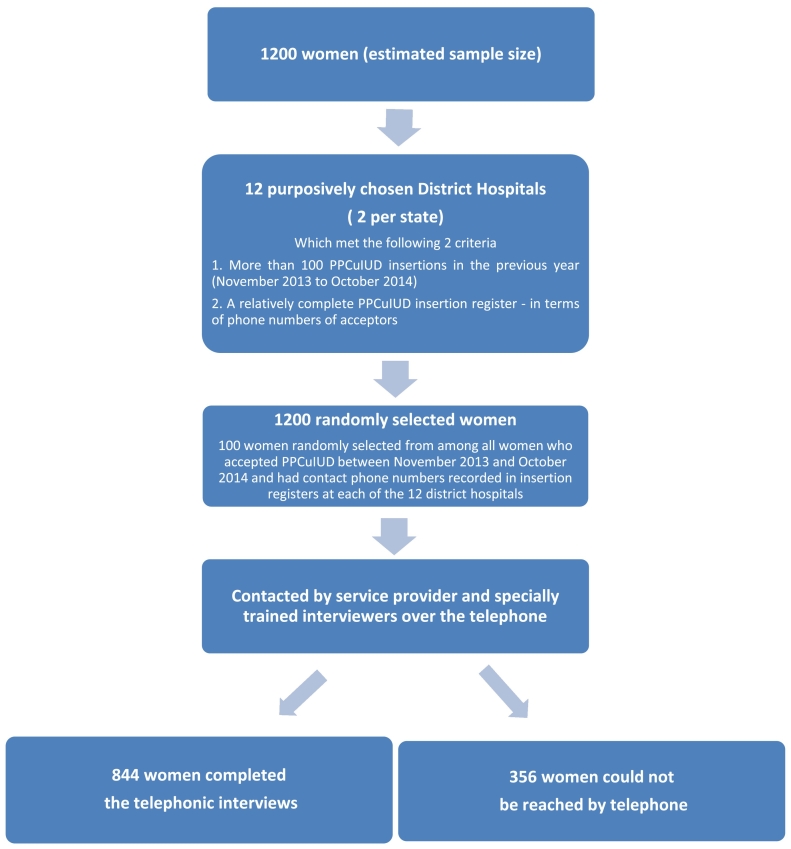
Flowchart depicting selection of study participants.

In each of the 6 states, we selected 2 district hospitals, that is, a total of 12 district hospitals (Fig. 1). We first made a list of district hospitals that reported more than 100 PPCuIUD insertions in the previous year (November 2013 to October 2014). A total of 178 district hospitals out of 227 met this criterion across the 6 states. We then purposively selected 12 of them, 2 per state, based on feasibility of carrying out the data collection as well as the regular practice of recording phone numbers of PPCuIUD acceptors in the PPCuIUD insertion registers. Family planning service providers at district hospitals maintain PPCuIUD insertion registers. All women had received a Copper T 380A.

At each hospital, we randomly selected records of 100 women from among all PPCuIUD insertions recorded with the telephone contact information for the women from November 2013 to October 2014. Staff at each hospital (usually FP counselors) initially provided us with the total number of insertions recorded during this period. We generated 100 random numbers within this total number using Microsoft Excel and shared these with the hospital staff. The staff pulled the corresponding client records and telephoned these randomly selected women to ask if they would be interested in participating in the study. If the women expressed interest, the hospital staff gave their telephone number to trained interviewers of a research agency.

### 2.2 Type of PPCuIUD insertions

PPCuIUD insertions, that is, insertions which happen within 48 h of delivery, are further categorized as postplacental insertions and early postpartum insertions. Postplacental insertion after vaginal or cesarean delivery refers to insertion performed within 10 min after expulsion of placenta, whereas early postpartum insertion is done after 10 min but within 48 h of delivery. Women who had decided on taking up PPCuIUD during their antenatal period underwent postplacental insertion, while those who opted for it after their delivery underwent early postpartum insertion. Doctors performed postplacental insertions after cesarean delivery, and both doctors and nurses performed postplacental insertions after vaginal delivery and early postpartum insertions.

### 2.3 Data collection procedure and measures

We used a pretested semistructured questionnaire for data collection. We pretested the questionnaire with a sample of 10 women from facilities other than the study sites after obtaining their oral consent. This was done prior to data collection and resulted in modifications to some of the questions, including rephrasing or permitting multiple responses to few questions. The authors trained the interviewers in using the questionnaire and study procedures for 2 days and carefully supervised them during data collection to ensure data quality.

Trained interviewers interviewed the women over telephone. The time interval between PPCuIUD insertion and telephonic interview ranged between 13 months and 25 months for study participants. Interviewers obtained an oral consent from the women at the beginning of the call and audio recorded the consent process. They gave women the option to skip questions they did not want to answer or stop the interview if they felt uncomfortable.

During phone interviews, the interviewers collected data on sociodemographic characteristics (age, number and sex of children, education, occupation, income and number of family members) and experience with the PPCuIUD (continued use, removal, expulsion, time interval between insertion and removal or expulsion, associated side effects, reason for insertion, reason for removal and switch to other FP method). The associated side effects (pain, bleeding and discharge) were clubbed as a single option in the questionnaire considering the feasibility of asking medical-history-related questions over the telephone. An updated BG Prasad's scale was used to calculate the socioeconomic status of women [23]. They recorded women's responses on paper copies of the questionnaire.

After the interviews, interviewers abstracted four items from the registers: type of insertion (postplacental insertion after vaginal delivery, postplacental insertion after cesarean delivery or postpartum), type of service provider who inserted the PPCuIUD (doctor or nurse), date of insertion and phone number.

### 2.4 Analysis

We entered data in Microsoft Excel, double checked all entries and analyzed it using Stata 13.0 (Stata Statistical Software: Release 13; StataCorp LP, College Station, TX, USA) software.

In the analysis, we assessed background characteristics of the respondents and PPCuIUD outcomes at 1 year. We also analyzed reasons for PPCuIUD removal and the contraceptive switching choices among respondents who discontinued use of PPCuIUD.

### 2.5 Ethical approval

Ekjut Institutional Ethics Committee, an India-based ethics committee, and the Institutional Review Board of Johns Hopkins Bloomberg School of Public Health gave ethical approval for the study. All state governments also gave their prior approval for conducting this study.

## 3 Results

Of the 1200 women selected randomly, the hospital staff reached 844; the remaining 356 (29.6%) women could not be reached by phone even after three attempts made on different days. A significantly higher proportion of selected women could be reached in Bihar when compared to other states.

All women who could be reached by hospital staff agreed to participate in the study. All women answered the main questions. Four women did not answer about the symptoms they experienced during PPCuIUD use.

### 3.1 Sample characteristics

Most (57%) women were aged 25 years or above, and 63% had a postplacental insertion after vaginal delivery. More than two thirds of the women had opted for PPCuIUD in order to space pregnancies. Nursing staff did more than half of the insertions (57.3%) (Table 1).

**Table 1:** Distribution of study participants who received PPCuIUD at sampled health facilities in 2013–2014 according to sociodemographic characteristics, provider type, type of insertion and associated side effects experienced (*n*=844).

Characteristic	Number (%)
State	
Uttarakhand	128 (15.2)
Madhya Pradesh	138 (16.4)
Chhattisgarh	131 (15.5)
Jharkhand	137 (16.2)
Bihar	188 (22.3)
Uttar Pradesh	122 (14.5)
Age of the client	
<25 years	364 (43.1)
≥25 years	480 (56.9)
Education of the client	
Illiterate/just literate	192 (22.8)
Up to 5th standard	117 (13.9)
Up to 12th standard	396 (47.0)
Graduate/postgraduate	139 (16.3)
Occupation of client	
Housewife	747 (88.5)
Working	97 (11.5)
Number of living children	
0	12 (1.4)
1	369 (43.7)
2	304 (36.0)
3 or more	159 (18.9)
Number of living male children	
0	257 (30.5)
1	452 (53.6)
2	113 (13.4)
3 or more	22 (2.6)
Reason for accepting PPCuIUD	
Spacing	593 (70.3)
Limiting	217 (25.7)
Don't know	34 (04)
Type of insertion	
Postplacental insertion after cesarean delivery	141 (16.7)
Postplacental insertion after vaginal delivery	532 (63.0)
Early postpartum	171 (20.3)
Type of service provider who inserted PPCuIUD^a^	
Doctor	359 (42.7)
Nurse	481 (57.3)
Socioeconomic status	
Lower class	108 (12.8)
Lower middle class	298 (35.3)
Middle class	222 (26.3)
Upper/upper middle class	216 (25.6)
Experienced pain in abdomen/bleeding/discharge^a^	
Yes	314 (37.2)
No	463 (54.9)
Not available (for those who reported expulsion)	63 (7.5)

a Data missing for four women.

### 3.2 PPCuIUD outcomes at 1 year by insertion type

The 1 year postpartum continuation rate was 62.8% (95% CI: 59.4%–66.1%), 1 year postpartum removal rate was 29.7% (95% CI: 26.7%–32.9%), and the 1 year postpartum expulsion rate was 7.5% (95% CI: 5.8%–9.4%). Of the 7.5% of women who experienced expulsion by 12 months, 4.7% reported expulsion occurred before 6 weeks, 2.1% reported expulsion between 6 weeks and 6 months, and only 0.7% reported expulsion after 6 months (data not shown). By insertion type, 1-year postpartum continuation rate was highest for early postpartum insertions [67.8% (95% CI: 60.2%–74.7%)], while 1-year postpartum expulsion rate was highest among women who had postplacental insertion after vaginal delivery [9.6% (95% CI: 7.2%–12.4%)] (Table 2).

**Table 2:** Distribution of outcomes of PPCuIUD insertion at 1 year among Indian women who received PPCuIUD in 2013–2014 according to type of insertion

Type of insertion⁎	Total	Continuation (%) (95% CI)	Removal (%) (95% CI)	Expulsion (%) (95% CI)
Postplacental insertion after cesarean delivery	141	66.7 (58.2–74.3)	29.1 (21.7–37.3)	4.2 (1.5–9.1)
Postplacental insertion after vaginal delivery	532	60.1 (55.8–64.3)	30.3 (26.3–34.3)	9.6 (7.2–12.4)
Early postpartum	171	67.8 (60.2–74.7)	28.7 (22.1–36.1)	3.5 (1.3–7.5)
Total	844	62.8 (59.4–66.1)	29.7 (26.7–32.9)	7.5 (5.8–9.4)

⁎ Postplacental insertion: done within 10 min after expulsion of placenta; early postpartum insertion: done after 10 min but within 48 h of delivery.

**Table 3:** Contraceptive method switching choices among Indian women whose PPCuIUDs were removed or expelled within 1 year of insertion (*n*=314)

Contraceptive methods	Removals Number of clients (percentage)	Expulsion Number of clients (percentage)
Condoms	70 (27.9)	16 (25.5)
Female sterilization	30 (11.9)	4 (6.3)
OCPs	29 (11.6)	5 (7.9)
IUCD	3 (1.2)	1 (1.6)
Injectable	1 (0.4)	1 (1.6)
Male sterilization	1 (0.4)	0
Others	3 (1.2)	4 (6.3)
No contraceptive used	114 (45.4)	32 (50.8)
Total	251 (100.0)	63 (100)

OCPs, oral contraceptive pills.

### 3.3 Reasons for removal and contraceptive method switching

Among women who had their PPCuIUD removed, almost two thirds (64.9%) cited associated side effects like bleeding, pain in abdomen and discharge as the primary reason for removal (Supplemental Table 1). Nearly half (46.5%) of the respondents whose PPCuIUDs were either expelled or removed did not change to any other modern contraceptive method. Majority of those who did change opted for condoms (Table 3).

## 4 Discussion

We found that two thirds of the women (62.8%) continue with their choice of PPCuIUD at 1 year postpartum. The 1 year postpartum removal and expulsion rates were 29.7% and 7.5%, respectively. Of those women who had their PPCuIUD removed before 1 year, two thirds cited associated side effects like bleeding, pain in abdomen and discharge as the primary reason. After discontinuation (removal and expulsion), almost half of the respondents did not switch to any other modern contraceptive method.

The PPCuIUD continuation rate in our study is comparable with findings of a study done in Turkey [24]. However, it is lower than rates reported by multiple other studies [25], [26], [27]. This difference should be seen in light of the fact that the results of our study may not be generalizable outside India and most of these prospective studies are single-hospital-based studies conducted in controlled hospital settings with smaller sample sizes. The results of our study however demonstrate that a well-planned and executed PPCuIUD program can achieve satisfactory level of continuation rates even when implemented on a large scale.

The expulsion rate at 1 year postpartum in our study (7.5%) is lower than that reported by another study done in Turkey [28] but higher than that reported (5.6% in the period of 6–12 months postpartum) in Zambia by Blumenthal [21]. The estimated expulsion rate of 4.7% until 6 weeks is comparable with the expulsion rate reported by a prospective study done in similar settings in India [3]. These expulsion rates need to be seen from the perspective that when PPCuIUD services were introduced in India in 2010–2011, there were widespread apprehensions due to high expulsion rates resulting from use of wrong insertion techniques. This was addressed through competency-based trainings with the use of a design-modified Kelly's placental forceps for PPCuIUD insertion — which led to expulsion rates of 4% at 6 weeks [3].

The PPCuIUD removal rate at 1 year is comparable to the discontinuation rates of other long-acting reversible contraceptives like levonorgestrel intrauterine systems and progestin implants [29]. A large proportion (45.4%) of women who got their PPCuIUD removed did not opt for any other contraceptive method after removal, while only 6% of the removals were due to the desire for another child. This lack of a switch to other spacing methods has been reported in another study done in similar settings [12]. It may be due to a lack of access to other spacing methods in the peripheral health facilities or a lack of awareness about other available methods among women. Another contributing reason could be limited contraceptive choices in India's public health system.

Discomfort due to bleeding, pain in the abdomen or discharge as main reasons for removal is consistent with findings of previous studies conducted in similar settings [12], [16], [19]. This finding emphasizes the need for quality pre- and postprocedure counseling for women so that they are aware that these side effects are to be expected and should not come as a surprise. Although we did not probe into the status of counseling in this study, earlier studies have reported that counseling is associated with increased continuation rates of IUCD [13], [18], [30].

The main strengths of our study are its size and that it has been conducted in a community-based resource-limited setting. It is a multicentric study conducted within a large-scale national program, which has seen more than 2.8 million insertions conducted across 2000 resource-constrained facilities over 6 years [7]. Therefore, our study findings are more representative of the “real-world settings.”

A major strength of our study is that it had no refusals for participation. Hospital staff — whom the women trust and tend to cooperate with — made the initial telephonic call for recruitment. They gave women the option of scheduling telephonic interviews as per their convenience. This facilitated participation and helped in avoiding refusals.

Our study also has some limitations relevant to the interpretation of the study findings. Recall bias may have affected our findings as study participants were interviewed 1 year after they chose PPCuIUD. Also, they were selected from among those PPCuIUD acceptors whose telephone contact numbers were recorded in the PPCuIUD insertion registers of health facilities. PPCuIUD acceptors without telephone numbers were not contacted, which could have led to a selection bias. In addition, purposive selection of the district hospitals and inability to telephonically contact around 30% of the randomly selected women may have led to a selection bias as well. We could not compare their sociodemographic characteristics with those of study participants, as this information was not recorded in the PPCuIUD insertion registers.

In conclusion, the results of this study show that PPCuIUD continuation rates at 1 year within the context of a large-scale national program are satisfactory. Expulsion rates are acceptable and comparable to the findings from other studies [3], [28]. The high number of removals due to discomfort caused by associated side effects of pain in abdomen, discharge and bleeding needs to be further addressed, as does the lack of uptake of other contraceptive methods after PPCuIUD discontinuation.

The following are the supplementary data related to this article.

Supplemental Table 1 Primary reason stated for removal of PPCuIUD by Indian women who received PPCuIUD in 2013–2014 and got it removed within one year of insertion Supplemental Table 1
