# Supplementary material for: One-year continuation of postpartum intrauterine contraceptive device: findings from a retrospective cohort study in India
Source: Contraception. 2019 Apr;99(4):212–6. doi: 10.1016/j.contraception.2018.12.003 (PMC6467543; doi:10.1016/j.contraception.2018.12.003)
Supplement: Supplemental Table 1 — Primary reason stated for removal of PPCuIUD by Indian women who received PPCuIUD in 2013–2014 and got it removed within one year of insertion [file mmc1.rtf]

Supplemental Table 1: Primary reason stated for removal of PPCuIUD by Indian women who received PPCuIUD in 2013-14 and got it removed within one year of insertion 

Reasons for removal	Number of clients (percentage)
	
Associated side effects*	163(64.9)
	
Husband/ family was against it	7( 2.8)
	
Wanted to have another child	15( 6.0)
	
Wanted to use some other contraceptive method
	25(10.0)	
Other reasons	41(16.3)
	
Total	251(100)	
*Includes bleeding, discharge, pain in abdomen
